# Digital Healthcare and Internet Derived Information Obstruction Treatment (IDIOT) Syndrome: Understanding the Link Between Clicks and Consequences

**DOI:** 10.7759/cureus.63438

**Published:** 2024-06-29

**Authors:** Jitendra Patel, Anamika Chakraborty Samant, Rainita R Pise, Hemali Jha

**Affiliations:** 1 Physiology, Gujarat Medical Education and Research Society Medical College, Vadnagar, IND; 2 Internal Medicine, Chaitanya Hospital and Obesity Centre, Mumbai, IND; 3 Community Medicine, Datta Meghe Medical College, Nagpur, IND; 4 Internal Medicine, Integral Institute of Medical Sciences and Research Centre, Lucknow, IND

**Keywords:** self-medication, self-diagnosis, behavior, online health information, idiot syndrome, digital healthcare

## Abstract

This article explores the phenomenon of Internet Derived Information Obstruction Treatment (IDIOT) syndrome, highlighting the impact of internet-derived health information on individuals’ treatment decisions. Drawing on recent studies, including the rise of IDIOT syndrome due to increased internet use and the potential risks associated with self-medication based on online information, the editorial emphasizes the importance of critically evaluating health information. Insights from research conducted in the last few years highlight the complexity of health conditions and the necessity of seeking professional medical guidance to address the various clinical conditions and their consequences. This article sets the stage for a detailed examination of the IDIOT syndrome and its implications for healthcare decision-making in the digital era.

## Editorial

The editorial on Internet Derived Information Obstruction Treatment (IDIOT) syndrome highlights the risks associated with individuals’ reliance on online health information for self-diagnosis and self-medication. It emphasizes the importance of critical evaluation of internet-derived health information to ensure accuracy, reliability, and relevance. The editorial underscores the growing influence of online resources on individuals’ treatment decisions, necessitating a cautious approach to avoid potential harm and misinformation. By discussing the implications of IDIOT syndrome and the significance of seeking professional guidance, the editorial aims to promote informed healthcare decision-making in the digital era.

The IDIOT syndrome, also known as “cyberchondria,” refers to the phenomenon where individuals may experience an increase in anxiety and engage in self-medication or abruptly cease prescribed treatments due to information obtained from the internet. This syndrome highlights the potential risks associated with relying on online health information without professional guidance, leading to adverse consequences for individual health outcomes.

The implications of IDIOT syndrome for healthcare decision-making underscore the importance of critically evaluating the reliability and accuracy of internet-derived health information. Individuals may be susceptible to misinformation, misinterpretation of symptoms, and inappropriate self-treatment practices, which can result in delayed or incorrect medical interventions. Healthcare providers play a crucial role in guiding patients toward evidence-based sources of information and promoting informed decision-making to ensure optimal health outcomes and safety. The growing influence of internet-derived health information on individuals’ treatment choices has become a significant trend in healthcare decision-making. With the widespread availability of online resources, individuals now have easy access to a vast array of health information, ranging from symptoms to treatment options. This accessibility has empowered patients to take a more active role in managing their health and seeking information about their conditions.

Li and Tang conducted a study that showed the significant impact of online health information-seeking behavior in shaping individuals’ perceptions of health conditions, including sexually transmitted diseases [1]. This infodemiological study highlights how internet searches can reflect public interest and concerns about specific health issues, influencing individuals’ decisions regarding disease prevention and management. Additionally, research by Vega et al. emphasizes that the accessibility of online health information has transformed patient decision-making processes [2]. While the internet offers opportunities for patients to educate themselves and engage in shared decision-making with healthcare providers, challenges related to the quality and reliability of online information remain. Patients may be influenced by misinformation or biased sources, influencing their treatment choices. The influence of internet-derived health information on treatment choices emphasizes the need for individuals to critically evaluate the information they encounter online and consult healthcare professionals for accurate diagnosis and treatment recommendations. Healthcare providers play a crucial role in guiding patients toward reliable sources of information and ensuring that treatment decisions are based on evidence-based practices rather than potentially misleading internet resources.

Risks of self-diagnosis and self-medication online

Online health information may not always be accurate or reliable, leading to the misinterpretation of symptoms and conditions. Individuals may misdiagnose themselves based on general information found online, which can result in inappropriate treatment or delays in seeking proper medical care [3]. Self-medication without professional guidance can result in individuals choosing inappropriate medications, incorrect dosages, or treatments that may not address the underlying health issue. This can lead to adverse drug reactions, drug interactions, or worsening of the condition [4]. Relying solely on online information for diagnosis and treatment may delay individuals from seeking timely medical advice from healthcare professionals. This delay can affect the effectiveness of treatment, especially in cases where early intervention is crucial for positive health outcomes [5]. Self-medication based on incomplete or inaccurate information obtained online may mask underlying health conditions or symptoms. This can result in temporary relief of symptoms without addressing the root cause of the problem, potentially leading to the progression of undiagnosed or untreated medical issues. These risks emphasize the importance of consulting healthcare providers for accurate diagnosis, appropriate treatment recommendations, and monitoring of health conditions. Healthcare professionals can offer personalized care tailored to individual needs, ensuring the safe and effective management of health concerns.

The rise of IDIOT syndrome can be influenced by various physical and psychological factors that impact individuals’ behavior and decision-making processes. The most important psychological factors, such as anxiety and uncertainty about one’s health condition, can drive individuals to seek quick solutions or reassurance online. The fear of a serious illness or the desire for immediate answers may lead to impulsive actions, including self-diagnosis and self-medication based on internet information. Individuals may exhibit confirmation bias, where they selectively seek and interpret information that aligns with their preconceived beliefs or concerns. This can lead to a distorted perception of health issues and a tendency to accept online information that confirms their existing worries, potentially exacerbating health anxieties. The accessibility of online health information can provide individuals with a sense of empowerment and control over their health decisions. While this can be empowering, it may also lead to overconfidence in self-diagnosis and treatment choices without considering the expertise of healthcare professionals. By understanding these psychological factors contributing to the rise of IDIOT syndrome, healthcare providers can tailor interventions to address patient anxieties, promote critical evaluation of online information, and foster collaborative decision-making between individuals and professionals for informed healthcare choices.

Research conducted in the last few years has highlighted the rise of the IDIOT syndrome, where individuals may self-medicate or abruptly stop prescribed treatments based on online information, leading to potential health risks. This underscores the need for a critical evaluation of internet-derived health information to prevent adverse outcomes. Furthermore, a study conducted in 2023 demonstrated the effects of online health information-seeking behavior on health decisions, particularly in the context of sexually transmitted diseases in China [1]. The study emphasized the influence of online resources on individuals’ perceptions of health conditions, indicating the importance of accurate and reliable information for informed decision-making. Additionally, a few past studies have explored how internet health information affects patient compliance in dermatology. The study revealed that patients often use online resources for self-diagnosis and treatment options, affecting the patient-physician relationship and treatment adherence. This highlights the complexities involved in navigating online health information and its implications for healthcare decision-making.

The importance of critical evaluation of online health information cannot be overstated in today’s digital age. With the abundance of health-related content available on the internet, it is crucial for individuals to assess the credibility, accuracy, and relevance of the information they encounter. Not all online health information is scrutinized by medical professionals or based on scientific evidence. Critical evaluation helps individuals distinguish between trustworthy sources and potentially misleading or inaccurate content, reducing the risk of misinformation. Each individual’s health condition and needs are unique. By critically evaluating online health information, individuals can ensure that the advice or recommendations align with their specific circumstances, promoting informed decision-making tailored to their well-being. Misinterpreted or incorrect health information can lead to harmful practices such as self-medication with inappropriate substances or delaying seeking professional help when needed. Critical evaluation can help individuals avoid potentially dangerous health behaviors. By emphasizing the importance of critical evaluation of online health information, individuals can empower themselves to make informed choices about their health, seek reliable sources of information, and collaborate effectively with healthcare providers for optimal health outcomes.

Future directions and clinical recommendations for digital literacy

Developing educational programs and campaigns to promote health literacy and digital literacy among the general population is crucial. These initiatives can focus on teaching individuals how to critically evaluate online health information, recognize reliable sources, and make informed healthcare decisions.

Enhancing telemedicine platforms to provide accessible and reliable virtual healthcare services is essential. Integrating telemedicine with educational resources can empower individuals to seek professional guidance online, reducing the likelihood of self-diagnosis and self-medication based on unreliable internet sources.

Creating tools or algorithms such as PageGraph, Attention-Based Deep Learning Model, eBizMBA, and Alexa Global Ranking Evaluation that assess the quality and credibility of online health information is essential for improving information reliability and guiding healthcare decisions. These tools can assist individuals in evaluating the trustworthiness of sources and help healthcare providers guide patients toward accurate and evidence-based resources.

Encouraging collaboration between individuals and healthcare providers to facilitate shared decision-making and open communication about online health information is crucial for promoting informed healthcare choices and improving patient outcomes. Healthcare professionals can play a pivotal role in guiding patients toward reliable sources and addressing any concerns or misconceptions related to internet-derived health information.

Conclusions

The concept of IDIOT syndrome sheds light on the potential challenges and risks posed by unchecked online health information. While the internet provides health-related resources, the dissemination of inaccurate, misleading, or unverified information can obstruct effective treatment and patient outcomes. It is imperative for healthcare providers, researchers, and policymakers to address the impact of IDIOT syndrome by promoting critical health literacy, enhancing the credibility assessment of online information, and encouraging patients to consult reputable sources for accurate medical guidance. By focusing from a future perspective, researchers and healthcare professionals can work toward mitigating the risks associated with IDIOT syndrome and promoting a more informed and empowered approach to healthcare decision-making in the digital age.
